# Exon-level estimates improve the detection of differentially expressed genes in RNA-seq studies

**DOI:** 10.1080/15476286.2020.1868151

**Published:** 2021-01-30

**Authors:** Arfa Mehmood, Asta Laiho, Laura L. Elo

**Affiliations:** a Turku Bioscience Centre, University of Turku and Åbo Akademi University, Turku, Finland; b Institute of Biomedicine, University of Turku, Turku, Finland

**Keywords:** RNA-seq, gene-level read counts, exon-level read counts, transcript-level read counts, transcript compatibility counts, differentially expressed genes

## Abstract

Detection of differentially expressed genes (DEGs) between different biological conditions is a key data analysis step of most RNA-sequencing studies. Conventionally, computational tools have used gene-level read counts as input to test for differential gene expression between sample condition groups. Recently, it has been suggested that statistical testing could be performed with increased power at a lower feature level prior to aggregating the results to the gene level. In this study, we systematically compared the performance of calling the DEGs when using read count data at different levels (gene, transcript, and exon) as input, in the context of two publicly available data sets. Additionally, we tested two different methods for aggregating the lower feature-level p-values to gene-level: Lancaster and empirical Brown’s method. Our results show that detection of DEGs is improved compared to the conventional gene-level approach regardless of the lower feature-level used for statistical testing. The overall best balance between accuracy and false discovery rate was obtained using the exon-level approach with empirical Brown’s aggregation method, which we provide as a freely available Bioconductor package EBSEA (https://bioconductor.org/packages/release/bioc/html/EBSEA.html).

## Introduction

RNA-sequencing (RNA-seq) technology is widely used in basic and applied research to study the transcriptome by enabling the quantification of all expressed genes in given samples simultaneously[1]. A typical goal in RNA-seq studies is to identify differentially expressed genes (DEGs) between sample groups representing, for example, different cell types, perturbations, or states, in order to understand the underlying biological mechanisms [2].

The computational methodologies to analyse RNA-seq data are still in the process of refinement. The conventional RNA-seq data analysis pipeline includes three main steps: 1) read alignment to the reference genome, 2) gene expression level quantification and 3) detection of DEGs. After read alignment, the expression level quantification is typically achieved using counting schemes that summarize the read counts across the exons for each gene, using exon intersection or exon-union methods. In the exon-union approach, reads from all exons across the different isoforms are summed, whereas in the exon-intersection, only the reads from constitutive exons – exons which are consistently conserved after splicing – are considered [2]. These gene-level read counts are then used as input for differential expression testing with methods such as DESeq2 [3], limma [4], edgeR [5], or ROTS [6] (see [7] for a comprehensive overview of differential expression testing methods).

Recently, we and others have demonstrated the benefits of using exon-level read counts or transcript abundances over the gene-level read counts for identifying DEGs [9–12].^8^ The suggested benefits of this alternate approach include increased power in statistical testing and diminished bias caused by the complex alternatively splicing events. With the conventional gene-level approach, it is difficult to detect differential gene expression reliably when the difference in the total read counts of a gene is small between the sample groups. In this case, the statistical power can be increased if a larger number of measurement points are available in the statistical testing, such as read counts across multiple exons. Due to the alternative splicing, it is also possible that the differential gene expression remains undetected if the expressional differences heavily vary across the different parts of the gene. This can be a significant limitation in the accurate detection of DEGs with the conventional gene-level read count-based approach as with many organisms, a large proportion of the genes undergo alternative splicing – for example, around 95% [13] in human.

Instead of using the gene-level summary counts, the initial statistical testing can be alternatively performed at a lower feature-level, prior to aggregating the result in the gene-level. Here, we tested how the usage of exon-level read counts, transcript-level read counts, or transcript compatibility counts (TCCs) compares with the conventional gene-level approach in two publicly available RNA-seq datasets. MicroArray Quality Control (MAQC) benchmark dataset [14] contains one brain sample and one universal human reference sample and was chosen as it had corresponding quantitative real-time polymerase chain reaction (qRT-PCR) validation data available for a large number of genes (840). Additionally, we selected a relatively deeply sequenced prostate cancer dataset [15] (>60 million reads per sample) that contains 14 tumours and 14 normal samples. For aggregating the lower feature-level *p*-values, two different methods were considered: Lancaster method [16] and empirical Brown’s method (ebm) [17]. Lancaster method performed superior to Fisher [18] and Sidak methods [19] in the aggregation of transcript-level *p*-values in a recent study [12], but (like Fisher and Sidak) it assumes independence of the observations and thus does not take into consideration the typical situation where there are several transcripts or exons that are associated with the same gene. Therefore, we also included ebm that allows considering dependence between features.

## Results

**Figure 1.**
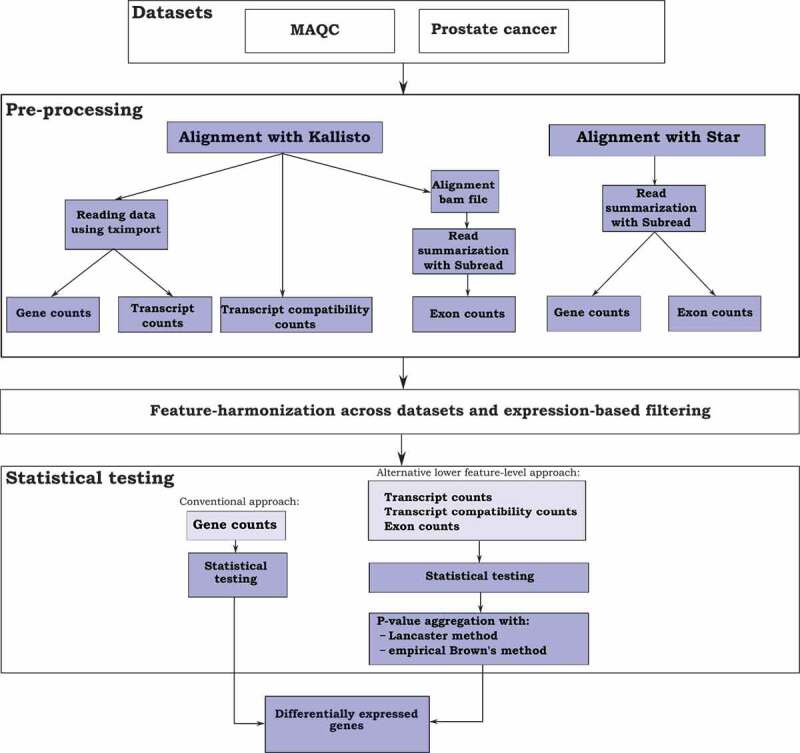
Schematic illustration of the different analysis steps. The two public datasets (MAQC and prostate cancer dataset) were preprocessed with either Kallisto- or Star-based workflow to produce gene-, transcript-, transcript compatibility- and exon-level read count data. After feature-harmonization across the datasets and expression-based filtering, statistical testing was performed at the gene-level or alternatively at the lower feature-levels in which case p-values were then aggregated to gene-level using two alternative approaches (Lancaster or empirical Brown’s method)

An overview of our comparison approach is shown in Fig. 1, summarizing the preprocessing steps to produce the count matrices at different feature levels and illustrating the difference in the statistical testing procedure based on the conventional and the proposed alternative approaches.

With the combination of Kallisto [20], Subread [21], and R/Bioconductor package tximport [22], it is possible to produce read counts for all the four different feature levels (gene, transcript, TCC, and exon). Additionally, the popular conventional analysis approach combining Star [23] alignments with Subread summarization to produce exon and gene-level read counts was run to see how these results compare with those based on Kallisto alignments.

Prior to statistical testing, the data at each feature-level were filtered and harmonized across the datasets in order to generate comparable results. In addition to removing very lowly expressed genes, genes with only one exon were filtered from all feature-level datasets as the results for them will not differ between the feature levels. The Ensembl annotation used contained 65,217 genes, of which 13,064 remained in the MAQC dataset and 13,998 in the full prostate cancer dataset after the harmonization and filtering.

Statistical testing at the different feature levels can be performed with any method available for RNA-seq data – here we used the popular DESeq2 R/Bioconductor package [3]. Transcript, TCC, and exon-level *p*-values were then aggregated to gene-level using Lancaster and empirical Brown’s method. The mean of normalized counts from the DESeq2 output was used as weights for the Lancaster method according to a previous example [12].

## Accuracy in MAQC dataset

**Figure 2.**
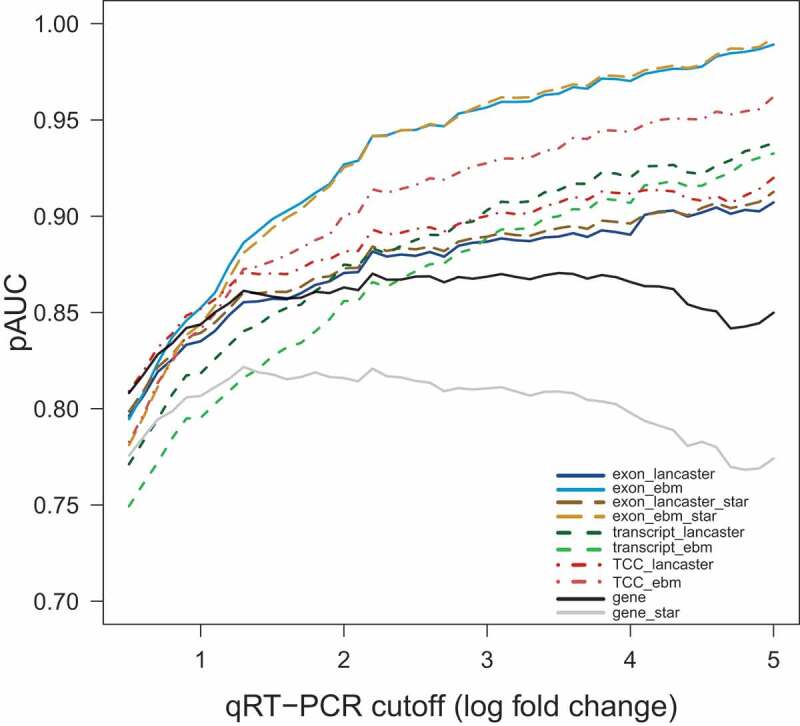
Partial area under the curve (pAUC) values at a specificity 0.8 at varying qRT-PCR cut-offs in the MAQC dataset, ranging from 0.5 to 5 with an increment of 0.1 across different analysis schemes

We investigated the accuracy of the different analysis schemes by calculating the partial Area Under the Curve (pAUC) for each scheme based on the qRT-PCR validation data available for the MAQC RNA-seq dataset. The pAUC for specificity above 0.8 was calculated at various log2-fold change cut-offs for the qRT-PCR data from 0.5 to 5, corresponding to 79–631 validated DEGs. The 97 genes with log2-fold change less than 0.2 in qRT-PCR were considered as true negatives. The results are summarized in Fig. 2, showing that all approaches using exon-, TCC- or transcript-based features outperformed the conventional gene-level analysis approach. Exon-based analysis approach with ebm for p-value aggregation had the best overall performance. Interestingly, with the Lancaster aggregation method, the exon-based approach performed worse than the other candidate approaches, indicating the importance of considering exon-dependence when working with exon-level read counts. The second best pAUC was reached with the TCC-based approach with ebm aggregation, and also, in this case, the result was much worse with Lancaster aggregation. Transcript-based approaches had mediocre performance, and with them, the difference between the aggregation methods was negligible. The Star-based analysis schemes at the exon-level provided very similar results with the corresponding Kallisto-based schemes. In contrast, the gene-level result was clearly worse with Star compared to the corresponding Kallisto-based result (Fig. 2).

## Number of DEGs and false discovery rate in prostate cancer dataset

**Figure 3.**
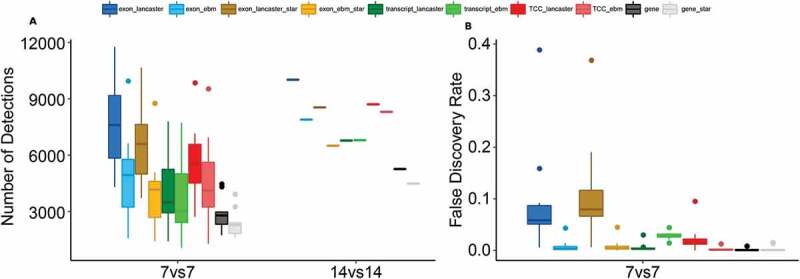
(A) Number of differentially expressed genes (DEGs) across different analysis schemes in the subset (7vs7) and the full prostate cancer dataset (14vs14) comparisons. (B) The false discovery rates across the analysis schemes based on mock comparisons

In the prostate cancer dataset, we studied the number of DEGs and false discovery rate (FDR) by means of investigating the number of DEGs in real and mock comparisons. In the real between-group comparisons, seven samples were randomly selected 10 times (without replacement) from both sample groups (tumour and normal). With Kallisto-based analysis schemes, the median number of DEGs in these subset comparisons ranged from 2660 (gene-level approach) to 7468 (exon-based approach with Lancaster aggregation) (Fig. 3A), whereas the full dataset resulted in 5127–9880 DEGs depending on the analysis scheme. More DEGs were generally detected with all the alternative approaches compared to the gene-level approach regardless of the choice of the aggregation method. Lancaster aggregation method provided more DEGs compared to the ebm at all feature levels, although the difference was subtler at the transcript-level.

To estimate the number of false positives (FPs), we performed a mock within-group analysis by sampling seven samples 10 times (without replacement) from the normal sample group into two groups. The genes found as differentially expressed in this within-group comparison were marked as FPs. FDR was then calculated by scaling the number of FPs (median number of genes detected in the 10 subset comparisons) by the number of DEGs found in the corresponding between-group comparison (Fig. 3B). Overall, the different analysis schemes showed low FDR, with the highest median FDR (0.06) observed for the exon-based analysis scheme with the Lancaster aggregation method, followed by the TCC-based scheme with Lancaster aggregation (FDR 0.01).

Star-based analysis schemes consistently provided a little fewer DEGs than the corresponding Kallisto-based analysis schemes, while FDRs remained similar (Fig. 3).

## Example genes in prostate cancer dataset

**Figure 4.**
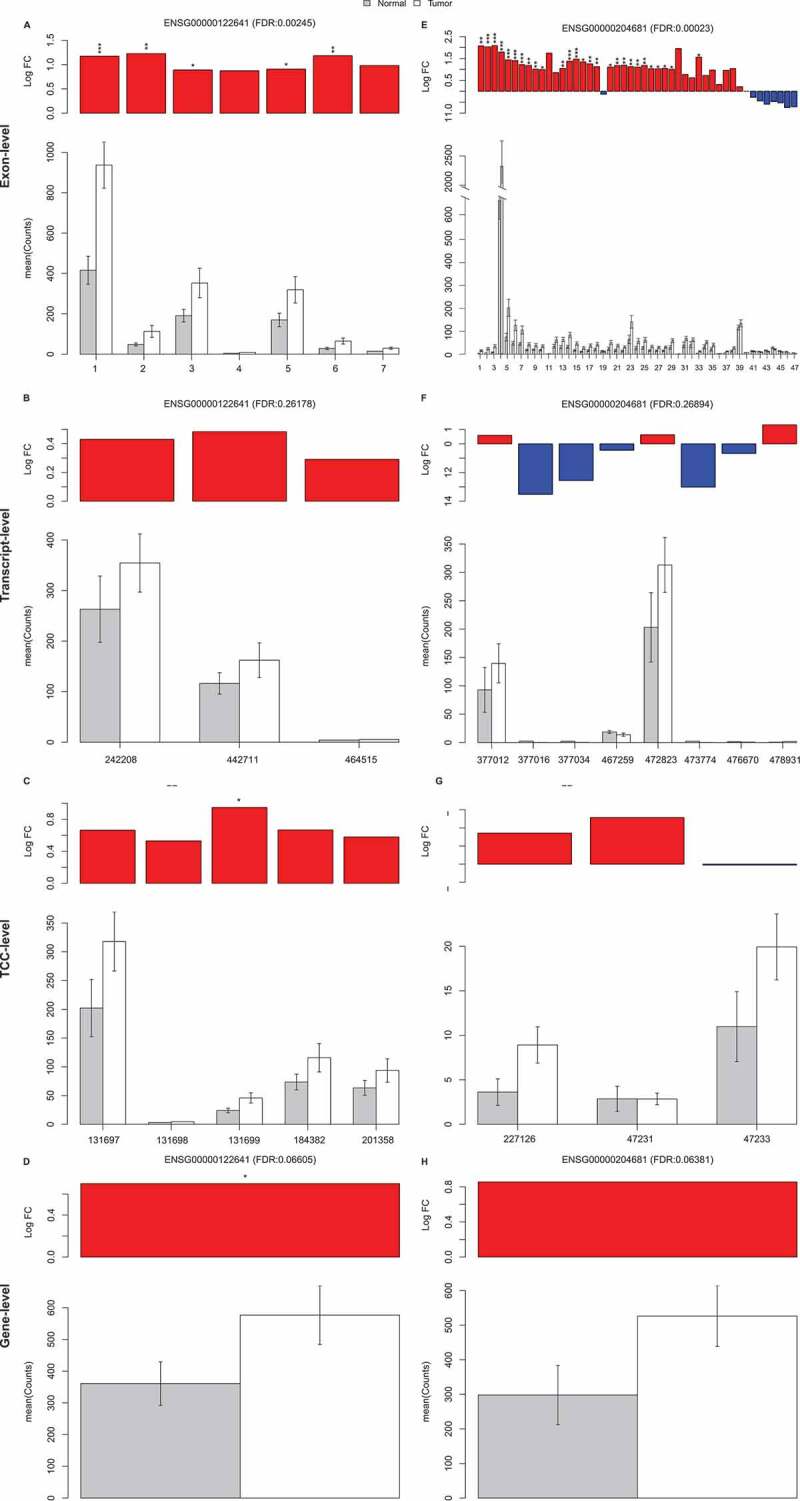
Visualization of two example genes that were detected significant (FDR < 0.05) between the tumour and normal samples in the full prostate cancer dataset by the Kallisto-based exon-level analysis scheme (with ebm aggregation) but not with the other feature-level analysis schemes. The genes are visualized in (A, E) at the exon-level, (C, G) at the TCC-level, and (B, F) at the transcript-level and (D, H) at the gene-level. The upper panel shows the log_2_ fold-change of each feature, asterisk indicating the *p*-value level (* *p* < 0.05, ** *p* < 0.01, *** *p* < 0.001). Ensembl gene identifiers and FDR are marked in the figure title. The lower panel shows the mean and the standard error of the normalized feature counts

The exon-based approach with ebm for *p*-value aggregation provided a large number of DEGs with reasonable FDR. Fig. 4 illustrates two representative examples of genes that were detected with this approach but missed by approaches based on other feature levels. Here, an expression level (normalized read count) and fold-change are visualized for each gene at the exon-level (A, E), transcript-level (B, F), TCC-level (C, G), and gene-level (D, H), along with the aggregated and feature-level statistical significance levels.

The first gene (ENSG00000122641, Fig. 4A-D) has seven expressed exons, five of which are significantly up-regulated. Although consistent up-regulation is evident at the exon-level, the difference in total counts at the transcript, TCC, or gene-level is not large enough to yield significant FDR for the gene with the analysis schemes based on these feature levels.

The second gene (ENSG00000204681, Fig. 4E-H) has 47 expressed exons, out of which 27 are significantly up-regulated. At the transcript, TCC, and gene-level, the partial up- and down-regulation cancel out one another, and the gene thus remains undetected.

In addition to illustrating how analysis of the data at a lower resolution signal level can improve the results, the examples shown also highlight the importance of visualizing the behaviour of the result genes at the exon-level in order to interpret the behaviour of the gene in a comprehensive way.

## Discussion

We investigated the accuracy and robustness of detecting the DEGs in RNA-seq studies using both the conventional approach based on the utilization of gene-level read counts and the alternative approach where the initial statistical testing is performed at a lower feature-level (transcript, transcript compatibility, exon), prior to aggregating the result to the gene-level. Two different aggregation methods were used: Lancaster and empirical Brown’s method (ebm), the latter being able to consider feature-dependence.

Earlier studies indicating the benefit of the alternative lower feature-level-based approaches over the conventional gene-level approach include the work by Liu et al. [24], where utilization of probe-level data improved the detection of DEGs in the context of microarrays [24]. With RNA-seq data, Laiho et al. [9] used the exon-level data and Yi et al. [11] transcript and TCC-level data to increase the accuracy and sensitivity of the differential gene detection. However, our study is, to our knowledge, the first one systematically comparing the use of several different feature levels and, in this context, investigating the effect of taking the dependence of features into consideration in statistical testing.

Kallisto has recently become a popular tool in RNA-seq data analysis and, with some auxiliary tools, allows the generation of count data matrices at all four feature levels, making it a convenient preprocessing tool for our study (Fig. 1). In addition, we generated exon- and gene-level count matrices based on Star alignments, representing the more traditional data preprocessing workflow. Our results suggested that the analysis schemes based on preprocessing with Kallisto generally performed slightly better compared to the analysis schemes based on Star alignments (Figs. 2 & 3). However, with the exon-based schemes, the difference was very small. Thus, for those looking for a simple analysis workflow, we recommend using the Star-based workflow at the exon-level.

Assessment of pAUC based on the MAQC RNA-seq dataset with complementary qRT-PCR validation data showed that the exon-based analysis scheme with ebm aggregation outperformed the other analysis schemes (Fig. 2). The same approach also showed good performance in the prostate cancer dataset, where it enabled the detection of a relatively high number of DEGs while showing low FDR (Fig. 3). Also, the TCC- and transcript-based analysis schemes improved the results over the conventional gene-level approach in both datasets. Thus, our results clearly show how the statistical power in detecting differential gene expression is increased due to the availability of several measurements per gene, consistent with the earlier studies [9,12].

Ebm aggregation provided a significant improvement to the results with the exon-based and TCC-based analysis schemes, while with the transcript-based schemes, the difference in the results between the two tested aggregation methods was negligible. This is likely to be explained by the ability of ebm to consider the dependence of features (several exons or transcripts associated with the same gene), most pronounced at the exon-level. The benefit of prohibiting the inflation of aggregated p-values by considering the feature dependence is also supported by the observation that with the exon-based analysis approach, the false discovery rate clearly elevated with the Lancaster method that ignores the feature dependence (Fig. 3). The fact that the transcript-based approach performed worse than exon- and TCC-based approaches indicates that estimating the transcript abundance still remains a challenging task. The downside with the TCCs, on the other hand, is that they are less intuitive to interpret biologically as different TCCs from the same gene do not have established annotations but are produced based on the data for each study. In fact, TCCs have originally been introduced to estimate the alternative splicing isoform frequencies and are calculated in the so-called equivalence classes, which consist of the reads that are compatible with the same set of transcripts [20].

Visualization of selected example genes illustrated how differential gene expression could be missed by the conventional gene-level analysis approach when the difference in total read counts is small or parts of the gene are regulated to different directions (Fig. 4) – a situation that easily arises in the context of alternative splicing. The visualizations also highlight the importance of inspecting the exon-level expression in order to validate and interpret the gene-level result. Thus, we provide an easy to use implementation of the superior differential gene expression analysis scheme (exon-based analysis with ebm aggregation) as a freely available EBSEA (Exon-Based Analysis for Expression) R/Bioconductor package (https://bioconductor.org/packages/release/bioc/html/EBSEA.html), coupled with exon-level expression visualization.

## Methods

### Datasets

The accuracy of the detected DEGs with the compared analysis schemes was accessed using RNA-seq data from the Microarray Quality Control (MAQC) project [14]. This experiment consists of two samples; one from Ambion’s human brain and another from Stratagene’s human universal reference RNA. The raw data are available at NCBI Short-Read Archive (SRA) under the accession number SRA010153. The corresponding qRT-PCR measurements for 840 genes, used as ground truth in our study, are available in Gene Expression Omnibus (GEO) under the accession number GSE5350. The second dataset referred to as the prostate cancer dataset, comes from a prostate cancer study [15] that contains RNA-seq data on tumour and normal samples (14 in each group) and is available from ArrayExpress under the accession number E-MAT-567.

### Data processing

The overview of the data processing workflow is described in Fig. 1. The raw RNA-seq data were processed in four alternative ways based on alignments or pseudo alignments from Kallisto tool [20] (v0.44.0) to produce the count data matrices at the different feature levels (gene, transcript, TCC, exon). In addition, gene and exon-level count matrices were also produced based on the alignments from Star aligner [23] (v2.6.1b). The human Ensembl-derived GRCh38 (release 80) genome and transcriptome annotation were used as a reference in the analysis. In the Kallisto-based workflows, the tool was run in quant mode to produce the transcript counts and in pseudo mode to produce the TCCs. R/Bioconductor package tximport [22] was used to produce the gene-level count matrix based on the transcript-level counts and description of gene models. With tximport, the gene-level counts are produced by summing the associated transcript counts. To obtain the TCC matrix from the result files from Kallisto, we used a script create_kallisto_ec_count_matrix.py from https://github.com/Oshlack/ec-dtu-paper/blob/master/create_kallisto_ec_count_matrix.py. The TCCs that mapped to different genes were removed before statistical testing. In order to generate the exon-level count matrix, Kallisto genomic alignment bam files were summarized with the Subread tool [21] (v.1.6.2). For this, gtf files containing the information on gene structure were flattened to remove the overlapping parts in the genes similar to a previous example [25].

With Star, the gene and exon-level count matrices were generated by summarizing the Star alignments with Subread to gene-level and separately to exon-level, using the exon-union approach.

Further analysis was performed using the R statistical language [26] (v 3.6.1) and the corresponding Bioconductor module [27] (v 3.10).

The data were normalized with relative log expression (RLE) method [3,28], and the datasets from different analysis schemes were filtered and harmonized to make them comparable. Filtering was performed by first removing features with normalized expression value less than one from each feature-level dataset (6 in total, see Fig. 1). After this, all genes with only one remaining exon at the exon-level were removed from all feature levels. Such genes were then removed from all feature-level datasets were, at any level, all the associated features had been removed.

### Statistical analysis

The statistical testing for all feature-level raw count matrices was performed using the DESeq2 (v1.26.0) [3] R/Bioconductor package. The feature-level p-values $p_{1},\ldots ,p_{K}$ were aggregated to gene-level using Lancaster [16] and empirical Brown’s methods (ebm) [17].

The Lancaster’s method is a generalization of the Fisher’s method, which is a commonly used method to aggregate *p*-values from K independent statistical tests. The test statistic of the Fisher’s method is

$$T_{Fisher}=\overset{K}{\underset{i=1}{\sum }}-2\log\left(p_{i}\right)$$

which follows a chi-square distribution with *2 K* degrees of freedom under the null hypothesis. The Lancaster’s method generalizes the Fisher’s method by introducing weights *w_1_*, … , *w_K_* to the *p*-values, assuming their independence. The test statistic becomes

$$T_{Lancaster}=\overset{i=1}{\underset{K}{\sum }}\Phi_{w_{i}}^{-1}\left(p_{i}\right)$$

which follows a chi-squared distribution with $\overset{K}{\underset{i=1}{\sum }}w_{i}$ degrees of freedom under the null hypothesis, where $\Phi_{w_{i}}^{-1}$ is the inverse cumulative distribution function of the chi-square distribution with *w_i_* degrees of freedom. In this study, the normalized mean counts were used as weights. The Fisher’s method is a special case of the Lancaster’s method when all the weights are set to 2 [12,29].

The empirical Brown’s method is an empirical adaptation of the Brown’s method [30], which is an extension of Fisher’s method to the case when the *p*-values are not independent [18]. Brown developed an approximation to the Fisher test’s null distribution when the *p*-values are derived from data from a multivariate normal distribution with a specified covariance matrix. It allows to consider the dependence of *k p*-values by using a rescaled chi-square distribution:

$$T_{Brown}c\chi_{2f}^{2}$$

where the constant *c* is a scale factor and *f* is the rescaled number of degrees of freedom. Brown showed that the covariance could be calculated by numerical integration. However, numerical integration is slow due to computational complexity and is not suitable for large datasets. Ebm is a non-parametric, empirical version of the Brown’s method, which approximates the covariance empirically directly from the data.

Finally, the *p*-values provided by the Lancaster’s method and ebm were corrected for multiple testing using the Benjamin Hochberg method. DEGs were called at gene-level FDR threshold of 0.05.

### Evaluation

The accuracy of the analysis workflows based on different feature levels and alignment strategies was evaluated based on the MAQC dataset that had qRT-PCT measurements available for 840 genes in addition to the RNA-seq results. pAUC for specificity above 0.8 was calculated using the pROC package (v 3.10) [31].

For the prostate cancer dataset, FDR was estimated by comparing the number of DEGs found in the within-group mock comparisons to those detected in the real between-group analysis. For the mock comparisons, seven samples from the normal sample group were randomly sampled without replacement 10 times, and the DEGs were called for each subset comparison. FDR was then calculated by dividing the median number of these false-positive findings to the number of detections in the between-group analysis.
